# Counseling Preferences Among Patients With Type 2 Diabetes: Implications for Personalized Care

**DOI:** 10.1155/jdr/9970845

**Published:** 2025-07-30

**Authors:** Thao Ngoc Phuong Nguyen, Chi Nguyen Thi, Trang Pham Thi Phuong, Quy Nguyen Ngoc, Hong Tham Pham

**Affiliations:** ^1^Department of Pharmacy, Nhan Dan Gia Dinh Hospital, Ho Chi Minh City, Vietnam; ^2^Department of Pharmacy, Nguyen Tat Thanh University, Ho Chi Minh City, Vietnam

**Keywords:** counseling, health education, Type 2 diabetes

## Abstract

**Background:** In Vietnam, diabetes-related knowledge and self-management practices remain suboptimal, with limited interventions addressing the diverse counseling needs of Type 2 diabetes (T2D) patients. This study is aimed at identifying the counseling topics most preferred by T2D patients and examine the factors influencing their preferences to inform the development of targeted, cost-effective health counseling initiatives.

**Methods:** A cross-sectional, descriptive study was conducted among 460 outpatients with T2D using structured interviews and medical records. Participants selected one of three counseling topics: disease-related knowledge (which provided information on the causes, symptoms, complications, and treatment options for T2D), nutrition and lifestyle (which included personalized guidance on food choices, healthy eating patterns, physical activity, and weight management), or medication information (which provided education on prescribed medications, dosage, timing, potential side effects, and proper administration). Multinomial logistic regression was employed to identify sociodemographic and clinical factors associated with counseling preferences.

**Results:** Among the participants, nutrition and lifestyle counseling was the most preferred topic (49%), followed by disease-related knowledge (33%), and medication information (18%). Factors influencing preferences included occupational status, and complications were significantly associated with a preference for disease-related knowledge (*p* = 0.021 and *p* = 0.029, respectively). Marital status and complications influenced the preference for nutrition and lifestyle counseling (*p* = 0.043 and *p* = 0.011, respectively). Medication regimen and achieving target fasting blood glucose levels predicted a preference for medication information counseling (*p* < 0.05 and *p* < 0.001, respectively).

**Conclusion:** This study serves as a reference for developing tailored health counseling programs to the specific needs of T2D patients. Personalized counseling approaches, particularly focusing on nutrition, lifestyle, and medication management, are critical for optimizing patient care and improving self-management. This study demonstrates that personalized counseling approaches, particularly in nutrition, lifestyle, and medication management, are critical for optimizing patient care and improving self-management. Additionally, it provides valuable insights for healthcare providers, policymakers, researchers, and stakeholders in implementing individualized care for T2D by considering specific factors that influence intervention choices.

## 1. Introduction

Diabetes is a serious, chronic condition that demands long-term management, placing a significant burden not only on patients but also on their families and society. The extended duration and complications of diabetes contribute to substantial healthcare costs. To enhance diabetes management and reduce economic strain, various interventions have been implemented, including dietary modifications and increased physical activity [1]. In 2024, the American Diabetes Association (ADA) emphasized the importance of the chronic care model, which identifies self-management support as one of its six key components [2]. Effective self-management is essential for diabetes care and requires consistent adherence to daily practices such as monitoring blood glucose levels, following a diet, maintaining foot care, meeting physical activity recommendations, and taking prescribed medications [3]. However, many patients still lack the necessary knowledge and skills for optimal self-care [4–6].

Studies have demonstrated that Type 2 diabetes (T2D) patients who engaged in adequate self-care activities achieved better glycemic control, experienced fewer complications, and reported improved quality of life [7]. Conversely, inadequate self-care is often associated with limited knowledge about diabetes management [8, 9]. In Vietnam, the levels of diabetes-related knowledge and self-management practices remain inadequate. Studies have shown that approximately 75% of the population has limited understanding of T2D [10] and only 8.5% of T2D patients are performing daily blood glucose monitoring at home [11]. Various interventions have been introduced to enhance patients' knowledge, treatment outcomes, and self-management abilities [12–14]. While T2D patients generally exhibited a positive attitude toward managing their condition [11, 15], their knowledge and practices were often insufficient, with minimal improvement over time [10, 11, 15]. This discrepancy between attitude and practical outcomes had been highlighted in previous research. For example, Asdaq reported that good knowledge did not always translate into a positive attitude or effective self-management [16]. A potential explanation for this finding was the lack of coordination and appropriateness in patient counseling program, which may fail to address patients' individualized needs.

Despite the recognized benefits of patient counseling, the implementation of comprehensive counseling sessions in Vietnam faces significant challenges. The high patient load and limited time available for healthcare providers, combined with economic constraints, make it difficult to deliver in-depth, multifaceted counseling on all aspects of diabetes care. Therefore, optimizing health education strategies is crucial to reduce costs and improve the efficiency of diabetes management programs. Vietnam was in urgent need of implementing health-promoting strategies [12, 17]. However, there was limited research on patients' specific counseling preferences and the factors influencing their choices. Most studies focus on the overall effectiveness of educational interventions rather than tailoring content to patients' needs [12, 18]. In resource-limited settings like Vietnam, understanding these preferences is crucial for optimizing diabetes counseling programs and improving patient engagement. This study is aimed at addressing these gaps by identifying the most preferred counseling topics among patients living with T2D and to examine the factors influencing their preferences. By exploring patients' informational needs, this research is not only aimed at improving patients' knowledge and practices but also at enhancing the quality of diabetes care while minimizing resource expenditure.

## 2. Materials and Methods

### 2.1. Study Design

A cross-sectional, descriptive study was conducted at Nhan Dan Gia Dinh Hospital, a tertiary general hospital with 4000 patient visits per day in Ho Chi Minh City, Vietnam, from January to September 2023. Our target population included outpatients diagnosed with T2D, aged 18 and above, based on their medical records. We excluded patients who were pregnant, had communication disabilities, cognitive or perceptual disorders, or lacked data on fasting blood glucose and HbA1c levels during the study period. Additionally, patients who did not require health counseling concerning T2D or those who refused to participate were also excluded. The study employed a convenience sampling method, where eligible participants were recruited based on their availability and willingness to participate during their outpatient visits. The study was reviewed and approved by the Ethics Committee of Nhan Dan Gia Dinh Hospital. All participants provided written informed consent voluntarily and had the right to decline participation at any time.

### 2.2. Data Collection Tools

Data from participants were collected through interviews and from their medical records at the time of recruitment. The interview questionnaire consisted of four parts. Part I collected the demographic characteristics of patients, including gender, age, occupational status, education level, marital status, and family medical history. Part II comprised behavior-related information, such as smoking and alcohol use, and support from healthcare staff. Part III comprised information related to clinical variables including body mass index (BMI), duration of illness, comorbidities, complications, current medication regimen, fasting blood glucose levels, and HbA1c levels using a pretested checklist. According to the ADA guideline dated on January 2023 [19] and the Guideline for Diagnosis and Treatment of T2D issued by Vietnam's Ministry of Health in December 2020 [20], patients were considered to have achieved the blood glucose target if their levels were in the range of 4.4–7.2 mmol/L. Achievement of HbA1c target was defined as level of below 6.5% (more stringent), 7%, or 8% (less stringent). Part IV included the assessment of patient counseling needs in three main specialized areas: disease-related knowledge, nutrition and lifestyle, and medication information. The participants were asked: “Which one of these counseling topics would you prefer to receive?” This allowed us to measure each patient's individual preference for counseling.

### 2.3. Health Counseling for Patients

**Figure 1:**
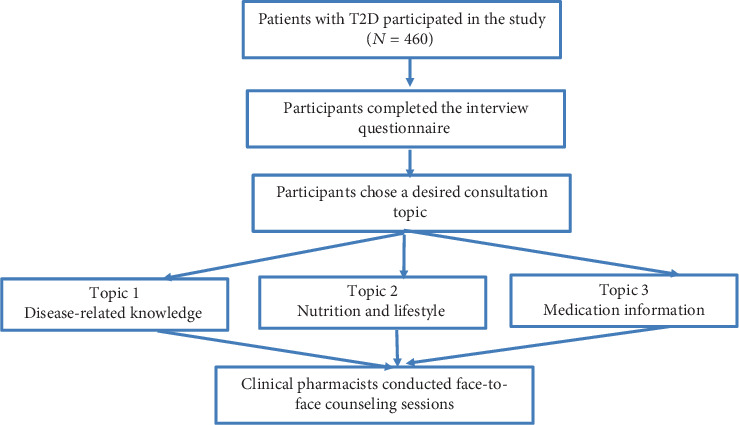
Flowchart of the study.

All in-depth counseling sessions were conducted by clinical pharmacists from the Department of Clinical Pharmacy at Nhan Dan Gia Dinh Hospital. Each patient had the option to voluntarily choose one of the three consultation topics in the health counseling program: disease-related knowledge, nutrition and lifestyle, and medication information. We provided in-depth and personalized counseling sessions based on the ADA guidelines (January 2023) [21, 22] and the Guideline for Diagnosis and Treatment of Type 2 Diabetes issued by Vietnam's Ministry of Health (December 2020) [20]. Each session lasted 20–30 min per patient and was conducted one-on-one (1:1). In the disease-related knowledge sessions, clinical pharmacists helped patients understand the causes, symptoms, complications, and treatment of diabetes. The importance of treatment adherence and self-monitoring of blood glucose at home was also emphasized during these sessions. In the nutrition and lifestyle sessions, patients received personalized guidance on food choices, healthy eating patterns, and suitable exercises tailored to their individual health condition. Strategies for maintaining a healthy weight and weight loss were also discussed. In the medication information sessions, patients were educated about the antidiabetic medications prescribed, including dosage, timing, potential side effects, and how to take properly. Stress management and anxiety reduction were also incorporated into these sessions. At the end of the consultation session, each patient received a handbook containing comprehensive information on diabetes, nutrition, physical activity, and insulin usage. The handbook was developed in collaboration with the Ngaydautien project (http://www.ngaydautien.vn) which has been previously utilized elsewhere [12].  Figure 1 illustrates the study design and data collection process.

### 2.4. Data Analysis Method

Data were entered and analyzed using Microsoft Excel and the R language (Version 4.4.2). Descriptive statistics were presented as frequency and percentage. To examine the impact of individual covariates on the likelihood of selecting disease-related knowledge or nutrition and lifestyle counseling topics compared to medication information, a multinomial logistic regression model was employed. All variance inflation factors (VIFs) were below 10, indicating no severe multicollinearity. The model adjusted for all other variables included. The statistical significance level was considered as 0.05 (*p* < 0.05).

## 3. Results

### 3.1. Sociodemographic Characteristics and the Counseling Needs in T2D Patients

Among the 460 participants in the study, more than half of the participants were female (54.1%) and under 60 years of age (52.6%). Most of the study population were not currently employed, including those who were unemployed and retired (72.6%), and most lived with relatives (88%). Additionally, nearly two-thirds of the participants reported having relatives with diabetes (62.8%) (Table 1).

With regard to clinical characteristics, approximately two-thirds of patients with T2D had uncontrolled HbA1c levels (60%), whereas the proportion of patients reaching the fasting blood glucose target was notably lower (39.6%). Interestingly, over 90% of the participants were classified as overweight or obese (BMI > 23 kg/m^2^; 98.3%). In terms of treatment regimens, more than 70% of patients were managed with oral hypoglycemic agents (OHAs) alone, followed by a combination of OHAs and insulin (23.3%). Only a small proportion of patients were treated with insulin alone (5.2%). Further details on the disease and therapy characteristics of the study population are presented in Table 2.

As shown in Table 3, most of the patients consumed no tobacco (92.0%). However, the number of patients who consumed alcohol was quite high (40%). The most surprising results were that less than 10% of patients received counseling support from the clinical pharmacist (9.8%), and nearly half of the participants did not receive any counseling support (47.8%) at the hospital.

The majority of individuals with T2D expressed a greater need for health counseling on nutrition and lifestyle (*n* = 227; 49%), followed by disease-related knowledge (*n* = 152; 33%) and medication information (*n* = 81; 18%).

### 3.2. Sociodemographic Factors Associated With the Counseling Needs in T2D Patients

In the multinomial logistic regression analysis (Table 1), only occupational status and marital status were statistically associated with counseling topic preferences. Occupational status was significantly correlated with the preference for disease-related knowledge counseling compared to medication information counseling (*p* = 0.021). Specifically, unemployed or retired individuals were more likely to choose disease-related knowledge counseling over medication information counseling compared to employed individuals (OR = 2.95, 95% CI: 1.18–7.40). However, there was no significant association between occupational status and the preference for nutrition and lifestyle counseling versus medication information counseling (*p* = 0.213). Marital status also showed a significant association with counseling topic preferences (*p* = 0.043). Patients living with a spouse were more likely to prefer nutrition and lifestyle counseling over medication information counseling (OR = 2.49; 95% CI: 1.03–6.01). While these patients also showed a tendency to prefer disease-related knowledge counseling over medication information counseling (OR = 1.56; 95% CI: 0.56–4.36), the results were not statistically significant (*p* = 0.398).

### 3.3. Behavior and Clinically Related Factors Associated With the Counseling Needs in T2D Patients

As presented in Table 2, a significant association was observed between clinically related factors and the choice of counseling topics. Disease complications were seen as a factor related to counseling topic preferences. Specifically, patients with complications were more likely to request counseling on disease-related knowledge compared to medication information (OR = 2.65, *p* = 0.029). A similar trend was observed for the nutrition and lifestyle topic, with patients exhibiting complications showing a higher preference for this theme over medication information (OR = 2.90, *p* = 0.011). Besides, medication regimen was another factor predicting the choice of counseling topics. Specifically, T2D patients treated with insulin alone or in combination with OHAs showed a higher likelihood of selecting medication information as their preferred counseling topic compared to disease-related knowledge (*p* = 0.013 for insulin alone; *p* < 0.001 for insulin+OHAs) and nutrition and lifestyle topics (*p* < 0.001 for insulin alone; *p* = 0.002 for insulin+OHAs). Similarly, patients who achieved their fasting blood glucose target were significantly more likely to prioritize counseling on medication information compared to nutrition and lifestyle counseling (*p* < 0.001).

## 4. Discussion

Nearly half of the patients in our study expressed a strong need for counseling on nutrition and lifestyle, while one-third prioritized counseling on T2D disease-related knowledge. However, due to constraints in human resources, material resources, and funding, we were only able to provide individualized counseling on one selected topic per patient, with usual care provided for the other topics. To the best of our knowledge, this is the first study to investigate personalized counseling preferences among T2D patients in Vietnam. Our findings highlight the significant importance of nutrition and lifestyle counseling. This result was consistent with the findings of Levina et al., who reported that Indonesian patients prioritized physical activity within chronic healthcare management program. Notably, participation rates in physical activity sessions were the highest among all components of the program, which required substantial investment, ranging from 2.1 to 5.8 million USD [23]. Vietnam is currently in the urgent need of implementing strategies to promote health through activities such as patients' clubs, workshops, and education sessions [12, 17, 24]. Several studies have demonstrated the effectiveness of these approaches in improving patient outcomes [12–14]. The finding of our study can serve as a valuable resource for healthcare managers, enabling them to assess and strategically invest in nutrition, diet, and physical activity counseling in terms of manpower and materials. Such investments could help optimize resource allocation and improve the cost-effectiveness of diabetes care in Vietnam.

The results of the multinomial logistic regression analysis indicated that patients most likely to benefit from disease-related knowledge counseling sessions were those who were unemployed or retired and those with complications. These finding suggested that personalized counseling needs for diabetes management were influenced by work status and the progression of the disease (e.g., the presence of complications). Previous studies in Vietnam have reported that more than 90% of the T2D population did not grasp the concept of the disease [10]. Work status has been identified as a significant factor associated with inadequate knowledge about diabetes mellitus [10, 15]. This may be explained by the economic challenges faced by unemployed or retired individuals, such as limited monthly income and the burden of healthcare expenses, which could restrict their access to educational resources. As such, additional efforts and support were required to improve knowledge about diabetes management. Furthermore, the presence of complications may also impact patients' understanding of the disease. For instance, complications affecting the eyes or other organs may act as barriers to accessing or fully engaging in educational programs [11]. To mitigate these barriers and slow the progression of complications, it is crucial to implement regular counseling and educational programs designed to enhance patients' knowledge, attitudes, and practices.

Marital status and the presence of complications were significant predictors of patients' preference for nutrition and lifestyle counseling. Patients who lived with family members or had complications were more likely to prioritize nutrition and lifestyle counseling as their primary need. Vietnamese adults with T2D adult patients were less likely to meet physical activity guidelines [25]. Previous studies have shown that support from family and friends can directly influenced self-management behaviors [26]. Moreover, spouses of individuals with T2D often have a positive influence on their partners' diet and nutrition. In Vietnamese culture, women typically take responsibility for choosing and preparing for the family. However, traditional Vietnamese diets often include high-glycemic index foods, such as white rice and sweetened condensed milk in coffee [27, 28]. Given this cultural context, healthcare providers should place greater emphasis on counseling not only for T2D patients but also for their family members, as they play a crucial role in encouraging healthy lifestyle and dietary habits. We believe that T2D patients in our study who lived with family members or had complications would benefit the most from nutrition and lifestyle counseling sessions, as supported by previous findings. For instance, one study reported that patients reduced their carbohydrate intake or switched to low-glycemic index foods after receiving dietary counseling from physicians [28].

Our analysis revealed that patients currently treated with insulin (alone or in combination with OHAs) and those who achieved fasting blood glucose targets were more likely to prioritize medication information as their primary topic of health counseling. This finding suggests that patients focused on managing their medications or achieving glycemic goals may place greater emphasis on acquiring detailed knowledge about their medications, including mechanisms of action, dosing schedules, timing of administration, potential side effects, and drug interactions. In Vietnam, nearly 50% of patients reportedly had good knowledge and practice regarding insulin use [29]. However, the rate of medication errors in the preparation and administration of insulin has been reported to reach approximately 30% [30]. In our study setting, many elderly patients expressed fear about self-administering insulin, particularly when lacking support and psychological care from healthcare workers and family members. Additionally, some T2D patients held a negative attitude towards insulin therapy, perceiving it as an indicator of the final stage of their disease, requiring lifelong dependence on injections. Previous research by Ngo, Vo, and Le demonstrated that patients who received counseling from healthcare professionals had significantly better knowledge of insulin administration (*p* = 0.001) [29]. These findings underscore the importance of addressing patients' fears regarding insulin use, particularly needle-related anxiety, whenever insulin was introduced into their treatment regimen. Moreover, Vietnamese patients commonly exhibit the fear of side effects associated with antidiabetic medications [31]. This has led to behaviors such as arbitrarily reducing dosages or discontinuing medication once their glycemic targets were achieved. Our finding highlighted the critical importance of providing medication-focused counseling for T2D patients, particularly those using insulin or achieving fasting blood glucose control. Tailored counseling interventions that address patients' specific concerns about medication management could enhance both their understanding and adherence, ultimately improving treatment outcomes.

### 4.1. Limitations of the Study

Our research had some limitations that need to be addressed. Firstly, the duration of data collection was relatively short, preventing us from evaluating the long-term effectiveness of the health counseling sessions postintervention. Secondly, as noted earlier, we were able to provide in-depth counseling on only one selected topic per patient, while other topics received only usual care due to constraints in manpower and material resources. Further research should explore the underlying reasons behind patients' counseling preferences and assess the effectiveness of personalized interventions in greater depth. Such studies would provide a more comprehensive understanding of how these factors influence patient outcomes and could inform strategies to optimize health counseling in diabetes management.

### 4.2. Implications for Practice

Our findings provide a reference for tailoring health counseling programs to address the diverse counseling needs of T2D patients. Given the high workload of endocrinologists and the limited availability of structured counseling sessions, the role of clinical pharmacists in patient education and counseling is becoming increasingly significant. These insights can guide policymakers and healthcare administrators in allocating resources effectively, ensuring adequate staffing, training, and material resources to support personalized counseling. Moving forward, greater interdisciplinary collaboration is needed to enhance the comprehensiveness of educational content. Regular evaluation of counseling programs and continuous professional development for healthcare workers will be crucial to adapt to the evolving needs of T2D patients, ultimately improving self-management and long-term outcomes. Strengthening these initiatives will help ensure that patients receive well-rounded, evidence-based education tailored to their individual needs.

## 5. Conclusion

Our study provides valuable insights into the counseling topics of greatest interest to T2D patients, highlighting the differing priorities in their informational needs. Nutrition and lifestyle were the most preferred counseling topics, followed by disease-related knowledge, while medication information was of least concern. Additionally, factors influencing the choice of counseling topics were clearly identified: occupational status and the presence of complications were associated with the desire for disease-related knowledge; marital status and complications were linked to interest in nutrition and lifestyle counseling; and the medication regimen and achievement of target blood glucose levels were the key determinants for interest in medication information. These findings also provide critical insights for healthcare providers, policymakers, researchers, and stakeholders in developing effective health education and counseling programs tailored to the needs of different patient groups.

## Figures and Tables

**Table 1:** Sociodemographic factors associated with the health counseling needs in T2D patients at Nhan Dan Gia Dinh Hospital (*N* = 460).

**Characteristics**		**Total** **N** = 460	**Medication information**	**Disease-related knowledge versus medication information**			**Nutrition and lifestyle versus medication information**		
		**n** ** (%)**	**n** = 81	**n** = 152	**OR (95% CI)**	**p** ** value**	**n** = 227	**OR (95% CI)**	**p** ** value**
Gender	Male	211 (45.9)	30	66	1	0.761	115	1	0.507
	Female	249 (54.1)	51	86	1.24 (0.31–4.90)		112	0.64 (0.17–2.37)	
Age groups (years)	≤ 60	242 (52.6)	48	76	1	0.373	118	1	0.420
	> 60	218 (47.4)	33	76	0.66 (0.27–1.63)		109	1.42 (0.61–3.34)	
Occupational status	Employment	126 (27.4)	32	34	1	**0.021**	60	1	0.213
	Unemployment/retirement	334 (72.6)	49	118	2.95 (1.18–7.40)		167	1.70 (0.74–3.94)	
Education level	Less than high school	230 (50.0)	38	85	1	0.834	107	1	0.206
	High school or more	230 (50.0)	43	67	1.10 (0.44–2.73)		120	1.75 (0.74–4.15)	
Marital status	Single/widowed/divorced	55 (12.0)	15	14	1	0.398	26	1	**0.043**
	Married/remarried	405 (88.0)	66	138	1.56 (0.56–4.36)		201	2.49 (1.03–6.01)	
Family medical history with T2D	No	171 (37.2)	26	66	1	0.188	79	1	0.605
	Yes	289 (62.8)	55	86	0.64 (0.32–1.25)		148	0.85 (0.46–1.58)	

*Note:* Values in bold indicate statistically significant differences (*p* < 0.05).

**Table 2:** Clinically related factors associated with the health counseling needs in Type 2 diabetes patients at Nhan Dan Gia Dinh Hospital (*N* = 460).

**Characteristics**		**Total** **N** = 460	**Medication information**	**Disease-related knowledge versus medication information**			**Nutrition and lifestyle versus medication information**		
		**n** ** (%)**	**n** = 81	**n** = 152	**OR (95% CI)**	**p** ** value**	**n** = 227	**OR (95% CI)**	**p** ** value**
BMI (kg/m^2^)	< 23	8 (1.7)	1	5	1	0.647	2	1	0.465
	> 23	452 (98.3)	80	147	0.47 (0.02–12.33)		225	0.30 (0.01–7.53)	
Duration of illness (years)	≤ 5	171 (37.2)	42	47	1	0.545	82	1	0.774
	> 5	289 (62.8)	39	105	1.28 (0.58–2.84)		145	0.90 (0.42–1.90)	
Comorbidities	No	15 (3.3)	7	2	1	0.191	6	1	0.070
	Yes	445 (96.7)	74	150	3.43 (0.54–21.67)		221	3.59 (0.90–14.27)	
Complications	No	292 (63.5)	64	91	1	**0.029**	137	1	**0.011**
	Yes	168 (36.5)	17	61	2.65 (1.10–6.34)		90	2.90 (1.28–6.58)	
Current medication regimen	OHAs	329 (71.5)	39	134	1	—	156	1	—
	Insulin	24 (5.2)	11	3	0.14 (0.03–0.66)	**0.013**	10	0.10 (0.03–0.31)	**< 0.001**
	OHAs+insulin	107 (23.3)	31	15	0.14 (0.06–0.31)	**< 0.001**	61	0.34 (0.17–0.68)	**0.002**
Reaching fasting blood glucose target	No	276 (60.0)	42	33	1	0.078	201	1	**< 0.001**
	Yes	184 (40.0)	39	119	2.56 (0.90–7.25)		26	0.15 (0.06–0.42)	
Reaching HbA1c target	No	182 (39.6)	39	28	1	0.153	115	1	0.903
	Yes	278 (60.4)	42	124	1.77 (0.81–3.87)		112	1.05 (0.51–2.15)	

*Note:* Values in bold indicate statistically significant differences (*p* < 0.05).

Abbreviation: OHAs: oral hypoglycemic agents.

**Table 3:** Behavior-related factors associated with the health counseling needs in T2D patients at Nhan Dan Gia Dinh Hospital (*N* = 460).

**Characteristics**		**Total** **N** = 460	**Medication information**	**Disease-related knowledge versus medication information**			**Nutrition and lifestyle versus medication information**		
		**n** ** (%)**	**n** = 81	**n** = 152	**OR (95% CI)**	**p** ** value**	**n** = 227	**OR (95% CI)**	**p** ** value**
Smoking	Nonsmoker	423 (92.0)	78	141	1	—	204	1	—
	Ex-smoker	23 (5.0)	3	4	1.02 (0.16–6.50)	0.986	16	1.38 (0.31–6.12)	0.676
	Current smoker	14 (3.0)	0	7	471,048.0 (215,902–1,027,717.2)	0	7	291,002.1 (133,379–634,899.2)	0
Alcohol use	No	276 (60.0)	56	95	1	0.428	125	1	0.736
	Yes	184 (40.0)	25	57	0.42 (0.65–7.61)		102	0.79 (0.20–3.10)	
Healthcare staffs supported	None	220 (47.8)	34	22	1	—	164	1	**—**
	Clinical pharmacists	45 (9.8)	5	37	0.48 (1.54–11.86)	0.285	3	0.33 (0.05–2.10)	0.240
	Others	195 (42.4)	42	93	0.35 (0.71–3.42)	0.877	60	0.66 (0.24–1.81)	0.420

## Data Availability

The data that support the findings of this study are available from the corresponding author upon reasonable request.
